# Editorial: Innovative *In Vitro* Models for Pulmonary Physiology and Drug Delivery in Health and Disease

**DOI:** 10.3389/fbioe.2021.788682

**Published:** 2021-10-22

**Authors:** Claus-Michael Lehr, Leslie Yeo, Josué Sznitman

**Affiliations:** ^1^ Helmholtz Center for Infection Research (HZI), Helmholtz Institute for Pharmaceutical Research Saarland (HIPS), Saarland University, Saarbrücken, Germany; ^2^ Department of Pharmacy, Saarland University, Saarbrücken, Germany; ^3^ School of Engineering, Royal Melbourne Institute of Technology, Melbourne, VIC, Australia; ^4^ Departments of Biomedical Engineering, Technion – Israel Institute of Technology, Haifa, Israel

**Keywords:** *in vitro*, organ on chips, lung on a chip, lungs, therapeutics, respiratory disease, drug screening and delivery

Respiratory diseases continue to exemplify a global leading cause of disability and mortality (Wisnivesky and De-Torres, 2019), with little signs of receding. In the midst of it, there is an ongoing and unmet need in treating such respiratory conditions. The situation has only been further exacerbated with the current COVID-19 pandemic and ensuing therapeutic challenges following the transmission of respiratory viruses (Scheuch, 2020; Leung, 2021; Wang et al., 2021). Concurrently, Chronic Obstructive Pulmonary Disease (COPD), with over 200 M patients, stands as one of the top five leading causes of death worldwide (Barnes and Stockley, 2005; Barnes et al., 2015a). All the meanwhile, acute respiratory distress syndrome (ARDS), acute lung injury (ALI), idiopathic pulmonary fibrosis (IPF) as well as other infectious diseases (e.g., pneumonia, tuberculosis) are either fatal diseases or pathologies exhibiting high mortality rates. Few new compounds that are safe have shown efficacy and have eventually emerged as new therapeutic options; rather, approved treatments have mainly consisted in improvements or repurposing of existing classes of drug (Barnes et al., 2015b), thus falling short of addressing existing the breadth of therapeutic needs. One critical issue lies in the existing discrepancy between the performance of therapeutic candidates in preclinical *in vivo* animal models and their high failure rate for safety and/or efficacy in reaching clinical trials. More generally, the efforts advocating for improved human-relevant *in vitro* lung models are intimately tied to current discussions on alternatives to *in vivo* animal experiments (Bonniaud et al., 2018) and have been further underlined with major hurdles faced with animal experiments regarding the extent to which these shed light on human pulmonary physiology and diseases (van der Worp et al., 2010; Benam et al., 2015; Artzy-Schnirman et al., 2021).

The present Special Issue exemplifies a timely snapshot of new research efforts aimed at delivering innovative and human-relevant pulmonary *in vitro* models, thereby overcoming the enduring disconnect between predictive capacities of pre-clinical *in vivo* animal models and novel respiratory therapeutics, as highlighted in the new review of Cidem et al. on the challenges and advances of *in vitro* models and the implementation of *ex vivo* inhaled drug screening models. In the field of *lung-on-chips*, this special volume first highlights new pulmonary platforms that are advancing more realistic *in vitro* inhalation assays with endpoints geared at assessing properties of the airway epithelium, including pharmacokinetics and barrier properties (Elias-Kirma et al.; Doryab et al.). Of particular interest, one new review covers advances in generating complex 3D culture systems that emulate the microarchitecture and pathophysiology of the human lungs using organotypic systems for host-environment and pathogen interaction (Jimenez-Valdes et al.). Concurrently, Yang et al. discuss in another review opportunities for *organ-on-chip* platforms to accelerate *in vitro* studies elucidating the cytotoxic effects of inhaled particulate matter (PM).

The Special Issue also exemplifies a number of state-of-the-art methodologies for research in respiratory physiology. This includes for example the use of high-precision cut lung slices subject to cyclic stretching in an effort to mimic physiological breathing (Mondoñedo et al.), in particular in cytotoxic studies on exposure to cigarette smoke, as well as the introduction of new fibroblast cell lines for *in vitro* disease modeling of pulmonary fibrosis at an air-liquid interface (Nemeth et al.). In parallel, Lingampally et al. summarize the current knowledge and limitations of strategies aiming to carry out methodical pre-clinical drug screening in pertinent *in vitro*, *ex vivo*, and *in vivo* models of pulmonary fibrosis, with a focus on relevant therapeutics that to date only reduce the expression of fibrotic markers. In recreating phenotypes of fibrotic lung tissue, Yamanishi et al. have combined a technique of printing microscale collagen gels embedded with fibroblast cells together with live cell imaging and automated image analysis to enable high-throughput analysis of the kinetics of cell-mediated contraction of this collagen matrix. Meanwhile, Hortsmann et al. describe a straightforward custom-made device, allowing connection to commercially available nebulizers with standard *in vitro* pulmonary cell culture plates for reproducibly depositing pre-metered doses of nebulized drugs. Finally, Majoral et al. have also aimed to develop and evaluate a new method using cascade impactor to measure particle size at human physiological temperature and humidity taking into account ambient air conditions.

To conclude, this volume highlights perhaps most importantly innovative proof-of-concept efforts geared at drug screening of potential drug candidates for pulmonary therapies. For example, Garcia-Mouton et al. focus on the potential use of pulmonary surfactant (PS) to deliver full-length recombinant human surfactant protein SP-D (rhSP-D) using the respiratory air-liquid interface as a shuttle, demonstrating that PS may transport rhSP-D long distances over air-liquid interfaces, and thus opening opportunities to empower the current clinical surfactants and surfactant replacement therapy (SRT). In parallel, Tang et al. have explored the anti-inflammatory properties of Poloxamer 188 (P188) in ischemia/reperfusion (IR)-induced acute lung injury models that can help to maintain plasma membrane function by suppressing multiple signaling pathways and maintaining cell membrane integrity. Finally, Wu et al. have explored the promising therapeutic potential of the dual pharmacological inhibition of two isoforms of rho-associated coiled-coil-forming protein kinase (ROCK 1 and 2) to counteract growth factor (TGF)-β-induced myofibroblast transformation and remodeling in mesenchymal-epithelial interactions that are known to contribute to chronic lung diseases (i.e., COPD, lung fibrosis) and defective lung repair.
